# Identification of SOX2 as a novel glioma-associated antigen and potential target for T cell-based immunotherapy

**DOI:** 10.1038/sj.bjc.6603696

**Published:** 2007-03-20

**Authors:** M Schmitz, A Temme, V Senner, R Ebner, S Schwind, S Stevanovic, R Wehner, G Schackert, H K Schackert, M Fussel, M Bachmann, E P Rieber, B Weigle

**Affiliations:** 1 Medical Faculty, Institute of Immunology, Technical University of Dresden, Dresden, Germany; 2 Medical Faculty, Department of Neurosurgery, Technical University of Dresden, Dresden, Germany; 3 Institute of Neuropathology, University Hospital Muenster, Muenster, Germany; 4 Avalon Pharmaceuticals, Germantown, MD, USA; 5 Department of Immunology, Institute for Cell Biology, University of Tübingen, Tübingen, Germany; 6 Medical Faculty, Department of Surgical Research, Technical University of Dresden, Dresden, Germany; 7 DKMS, Life Science Lab GmbH, Dresden, Germany; 8 Eucodis GmbH, Vienna, Austria

**Keywords:** brain tumour, tumour-associated antigen, glioblastoma multiforme, immunotherapy, SOX2

## Abstract

Prognosis for patients suffering from malignant glioma has not substantially improved. Specific immunotherapy as a novel treatment concept critically depends on target antigens, which are highly overexpressed in the majority of gliomas, but the number of such antigens is still very limited. *SOX2* was identified by screening an expression database for transcripts that are overexpressed in malignant glioma, but display minimal expression in normal tissues. Expression of *SOX2* mRNA was further investigated in tumour and normal tissues by real-time PCR. Compared to cDNA from pooled normal brain, *SOX2* was overexpressed in almost all (9 out of 10) malignant glioma samples, whereas expression in other, non-malignant tissues was almost negligible. SOX2 protein expression in glioma cell lines and tumour tissues was verified by Western blot and immunofluorescence. Immunohistochemistry demonstrated SOX2 protein expression in all malignant glioma tissues investigated ranging from 6 to 66% stained tumour cells. Human leucocyte antigen-A^*^0201-restricted SOX2-derived peptides were tested for the activation of glioma-reactive CD8+ cytotoxic T lymphocytes (CTLs). Specific CTLs were raised against the peptide TLMKKDKYTL and were capable of lysing glioma cells. The abundant and glioma-restricted overexpression of SOX2 and the generation of SOX2-specific and tumour-reactive CTLs may recommend this antigen as target for T-cell-based immunotherapy of glioma.

Malignant glioma represents the most common type of primary brain tumours in the United States (Hofman *et al*, 2006). Despite aggressive treatment with surgical resection followed by radiotherapy and chemotherapy, these tumours ultimately recur (Butowski *et al*, 2006). The median survival of patients suffering from glioblastoma multiforme (GBM), the highest-grade malignant astrocytoma, has not improved significantly over the past decades, remaining at about 12 months (Legler *et al*, 1999). The infiltrative nature of the tumour, the impracticality of optimal resection and the comparative intolerance of the normal brain for cytotoxic therapies together lead to a 5-year survival rate that is below 2% (Surawicz *et al*, 1998). This extraordinary high morbidity has triggered a desperate search for novel and more specific therapeutic approaches.

The use of either antibody-based or T-cell-mediated immunotherapy to selectively kill remnant glioma cells that could not be completely removed by surgery because of the infiltration of the tumour into the surrounding brain tissue has received increasing attention. Several animal models suggested that GBM may be amenable to immune therapeutic approaches (Sampson *et al*, 1996; Okada *et al*, 1998) and the identification of humoral as well as cellular immune responses in brain tumour patients triggered efforts to activate the immune system against glioma cells (Yu *et al*, 2001; Ueda *et al*, 2004). Although some encouraging pilot studies and clinical trials were reported, so far immunotherapeutic approaches were obviously hampered by the lack of defined target antigens (Ehtesham *et al*, 2004). Therefore, dendritic cells (DCs) pulsed with crude tumour cell homogenates or undefined peptide mixtures eluted from tumour cells were frequently used to augment glioma-reactive CTLs (Rutkowski *et al*, 2004; Yu *et al*, 2001, 2004; Yamanaka *et al*, 2003, 2005). However, to reduce the potential risk of destructive autoimmune reactions, it seems favourable to use groups of well-defined human leucocyte antigen (HLA) class I binding peptides derived from glioma-associated antigens that have been demonstrated to serve as target structures for T cells rather than introducing undefined mixtures of antigens to DCs.

To identify novel genes selectively expressed in malignant gliomas and to increase the very limited number of characterised glioma-associated CTL epitopes, we screened a large DNA chip-based expression database (Weigle *et al*, 2004). We found that the transcription factor *SOX2* was overexpressed in the majority of GBM samples whereas expression in normal brain and other non-malignant tissues was almost negligible.

*SOX* (*SRY*-like HMG box) genes represent a family of transcriptional cofactors implicated in the control of diverse developmental processes (Wegner, 1999). So far, more than 20 *Sox* genes have been described in mammals and are divided into six distinct groups according to their HMG-box homology (Schepers *et al*, 2002). *Sox* genes exhibit highly dynamic expression patterns during development of diverse tissues and cell types, especially during embryogenesis. In early stages of mouse and chicken embryonic development, *Sox*1–3 are highly expressed within the CNS and are downregulated as neural cells exit the cell cycle and start to differentiate (Uwanogho *et al*, 1995; Kamachi *et al*, 2000). In humans, *SOX2* mutations cause bilateral anophthalmia, a rare and severe form of malformation of the eye (Fantes *et al*, 2003). A number of *SOX* genes are amplified or upregulated in different tumours and tumour cell lines (reviewed in Dong *et al*, 2004).

In the present study, we demonstrated the selective overexpression of *SOX2* in the vast majority of malignant gliomas on the mRNA level as well as on the protein level. In immunohistochemical analyses, all GBM specimens investigated were positive for SOX2 protein, whereas SOX2 was not detectable in normal cortex. In addition, we identified an HLA-A^*^0201-restricted peptide derived from SOX2, which proved to be effective in activating tumour-directed CTL responses.

## MATERIALS AND METHODS

### Dot blot analysis

A 112 bp fragment of the *SOX2* cDNA corresponding to nt 1433–1544 of GenBank sequence NM_003106 was amplified from GBM cDNA with the primers SOX2_N3 (5′-AAATGGGAGGGGTGCAAAAGAGGAG-3′) and SOX2_C3 (5′-CAGCTGTCATTTGCTGTGGGTGATG-3′) using the following thermal profile: 95°C for 5 min; 40 cycles: 95°C for 30 s, 70°C for 30 s, 68°C for 30 s followed by a final extension at 68°C for 5 min. The PCR product was cloned into pCRII-TOPO (Invitrogen, Karlsruhe, Germany) and sequenced. The insert was isolated after *Eco*RI digestion, labelled with ^32^P (Megaprime DNA labelling system; Amersham Biosciences, Freiburg, Germany) and hybridised to a Human Multiple Tissue Expression (MTE) Array 2 (BD Clontech, Heidelberg, Germany). Hybridisation was performed according to the provider's instructions using 2.1 × 10^6^ c.p.m. ml^−1^ of the radioactively labelled probe, and signals were visualised by phosphoimaging (Amersham Biosciences).

### Brain tumour patients, tissue samples and cell lines

Primary tumour samples were obtained from brain tumour patients with informed consent and were intraoperatively frozen in liquid nitrogen (for RNA isolation) or fixed in 4% formaldehyde (for histology). For immunohistochemical analysis of SOX2 expression in normal cortices, tissue samples from autopsies verified to be free of disease were obtained from the Institute of Neuropathology, University Hospital Muenster (Muenster, Germany). Tissue specificity of mRNA expression was analysed by a quantitative PCR assay (see below) in three panels of normalised cDNAs derived from 16 normal adult tissues (Human MTC Panels I and II) and eight fetal tissues (Human Fetal Panel; all BD Clontech). In these panels, cDNA is pooled from several Caucasian individuals for each tissue type, including normal brain. The cDNA sample ‘glioma BC’ was from Biocat (Biocat GmbH, Heidelberg, Germany) and was characterised as a glioma from a male patient (catalogue no. C8235534, lot no. A605336).

The HLA-A^*^0201-positive mutant cell line T2, the chronic myelogenous leukaemia cell line K562 and the glioma cell line U373 (all from American Type Culture Collection, Manassas, VA, USA) were cultured according to the provider's instructions. The melanoma cell line 93.04A12.1 was kindly provided by Dr CJM Melief (University Hospital, Leiden, The Netherlands) and the glioma cell line U343 was a generous gift from Dr H Fischer (German Cancer Research Centre, Heidelberg, Germany). The HLA-A2-positive primary GBM cell preparations DD-HT4 and DD-HT6559 were maintained in DMEM supplemented with 20% FCS, 100 U ml^−1^ penicillin, 100 *μ*g ml^−1^ streptomycin, 4% (v v^−1^) non-essential amino acids (Biochrom, Berlin, Germany), and 50 *μ*g ml^−1^ gentamycin.

### RNA isolation and cDNA synthesis from tissue samples and cell lines

Total RNA was extracted by standard procedures (TriPure Reagent; Roche Diagnostics, Mannheim, Germany) and treated with DNase I (Amersham Biosciences). As *SOX2* is a single-exon gene, absence of traces of genomic DNA was checked by PCR before reverse transcription using *β-actin*-specific primers Act_N1 (5′-GCCGTCTTCCCCTCCATCGTG-3′)/Act_C1 (5′-GGAGCCACACGCAGCTCATTGTAGA-3′) with the thermal profile: 95°C for 3 min; 35 cycles: 95°C for 30 s, 70°C for 30 s, 68°C for 30 s followed by one round at 68°C for 7 min. The cDNA synthesis was performed using 1 *μ*g of total RNA and oligo-dT primers in a standard 20-*μ*l reaction (Advantage RT-for-PCR Kit; BD Clontech).

### Quantitative reverse transcription-PCR

Tissue specificity of *SOX2* mRNA expression was analysed by a quantitative light cycler (LC)-based PCR assay screening panels of normalised cDNAs derived from 16 normal adult tissues (Human MTC Panels I and II) and eight fetal tissues (Human Fetal Panel; all BD Clontech) as well as cDNAs from tumour samples and cell lines. The *SOX2* mRNA quantity was determined by an SYBR Green I-based real-time PCR protocol (LC – FastStart DNA Master SYBR Green I; Roche Diagnostics) using the primer pairs SOX2_N3/SOX2_C3 (see above). The PCR protocol for the *SOX2* LC assay consisted of a pre-denaturation step (10 min at 95°C) and 40 amplification cycles (15 s at 95°C, 5 s at 70°C, 12 s at 72°C). Specificity of the assay was checked by cloning and sequencing of the PCR product and by melting curve analysis. To quantify the transcript levels in specimens from brain tumour patients as well as in the cell lines U373 and U343, 2 *μ*l of the 1 : 5 diluted cDNA products was used for amplification with primer pair SOX2_N3/SOX2_C3.

The amount of *SOX2* transcripts was normalised to the quantity of *β-actin* transcripts. SYBR Green I-based quantification of *β-actin* was performed using the primers Act_N1 (5′-GCCGTCTTCCCCTCCATCGTG-3′) and Act_C1 (5′-GGAGCCACACGCAGCTCATTGTAGA-3′) applying the same PCR protocol as used for *SOX2*.

Serial dilutions of plasmid DNA containing the *SOX2* and *β-actin* fragments over eight log scales (10^1^–10^8^ molecules per capillary) were used as internal template standards (calculation via LC quantification software version 3.5; Roche Diagnostics). Each determination was carried out twice for each cDNA sample as independent PCR runs and molecule ratios of *SOX2* to *β-actin* transcripts were calculated from the mean values.

### Indirect immunofluorescence analysis

The glioma cell lines U373 and U343, the primary GBM cell preparations DD-HT4 and DD-HT6559 as well as the melanoma cell line 93.04A12.1 were grown on cover slides and fixed in ice-cold paraformaldehyde for 20 min. After washing in phosphate-buffered saline (PBS), cells were permeabilised with 1% sodium citrate/0.1% Triton X-100 and washed three times with PBS containing 0.1% bovine serum albumin (BSA). Cells were incubated for 1 h at room temperature with monoclonal anti-SOX2 antibody (MAB2018, R&D Systems, Wiesbaden, Germany, dilution 1 : 200). After washing again with PBS/0.1% BSA, the cells were incubated for 1 h at room temperature with Cy3-conjugated anti-mouse IgG (stock solution, 1 : 50 diluted, as recommended by the supplier, Dianova, Hamburg, Germany). After intensive washing with PBS the DNA was counterstained with Hoechst33342. Cells were examined by fluorescence microscopy (Olympus IX70, Hamburg, Germany).

### Immunoblot analysis

For protein analysis of SOX2 expression in the melanoma cell line 93.04A12.1 and the glioma cell lines U373 and U343, total protein lysates were prepared. Equal amounts of protein samples were subjected to electrophoresis and blotted onto PVDF membranes (PALL, Dreieich, Germany). SOX2 immunostaining was performed with the monoclonal antibody MAB2018. Equal loading of protein samples was confirmed by probing the blotted PVDF membranes with an anti-*α*-tubulin antibody (clone DM 1a, diluted 1 : 500; Sigma, Taufkirchen, Germany). The secondary goat anti-mouse antibody coupled to horseradish peroxidase (both diluted 1 : 2500, Dako, Hamburg, Germany) was visualised by using 3,3′-diaminobenzidine (DAB) (Sigma).

### Immunohistochemistry of glioma tissue

Immunohistochemistry was performed on paraffin-embedded human tumour tissue, diagnosed as glioblastoma (grade IV astrocytoma; WHO), according to the World Health Organization Classification (Kleihues and Cavenee, 2000). Paraffin-embedded cortex tissue from autopsies without pathological findings in the brain served as controls. Two micrometer paraffin sections were treated with Target Retrieval Solution pH 9.0 (Dako) for 35 min in a steamer for unmasking epitopes. The anti-SOX2 antibody MAB2018 was used in a concentration of 10 *μ*g ml^−1^ and incubated overnight at 4°C. Detection was performed with a biotinylated secondary anti-mouse IgG antibody BA-2001 (diluted 1 : 100, Vector Laboratories, Burlingame, CA, USA), using the avidin–biotin–peroxidase technique with DAB (Dako) as chromogen. Tissue was counterstained with haematoxylin. The proportion of SOX2-positive cells was determined by counting all cell nuclei as well as nuclei stained for SOX2 in three randomly selected high-power fields (=400-fold magnification) in the tumour core of each sample.

### Epitope prediction and peptide synthesis

Potential HLA-A^*^0201 ligands were selected from the amino-acid sequence of SOX2 using a matrix pattern suitable for the calculation of peptides fitting to an HLA-A^*^0201 motif (Rammensee *et al*, 1999); http://www.syfpeithi.de). The three highest scoring peptides were synthesised as described previously (Kiessling *et al*, 2002).

### Competition assay

Binding studies of potential HLA-A^*^0201-fitting peptides were carried out using the B-cell line JY and a fluorescence-based competition assay, essentially as described (Van der Burg *et al*, 1995), but without performing acid strip. Reporter peptide was ILK(FITC)EPVHGV from HIV-1 reverse transcriptase and positive control was YLLPAIVHI from RNA helicase p72. Fluorescence intensities were recorded by flow cytometry.

### *In vitro* generation of SOX2-specific CD8+ cytotoxic T lymphocytes

Briefly, peripheral blood mononuclear cells were prepared from blood samples of healthy donors by Ficoll–Hypaque (Biochrom, Berlin, Germany) density centrifugation. Monocytes were isolated by immunomagnetic cell separation with an anti-CD14 antibody coupled to paramagnetic microbeads (Miltenyi Biotech, Bergisch Gladbach, Germany) according to the manufacturer's instructions. Mature monocyte-derived DCs were generated as described previously (Kiessling *et al*, 2002). To generate SOX2-specific CTLs, mature DCs were pulsed with the SOX2-derived peptides 60029, 60030 and 60031 at a concentration of 20 *μ*g ml^−1^ of each peptide in serum-free RPMI 1640 medium for 3 h. After washing, 2 × 10^5^ peptide-loaded DCs were co-cultured with 2 × 10^6^ immunomagnetically isolated CD8+ T cells per well of a 24-well tissue culture plate (Greiner, Frickenhausen, Germany). T cells were cultured in 2 ml RPMI 1640 medium per well supplemented with 10% human serum (CCpro, Neustadt, Germany), 100 U ml^−1^ interleukin-2 (IL-2) and 10 ng ml^−1^ IL-7 (both from Strathmann Biotech, Hannover, Germany). Seven days later, cultures were washed and restimulated with peptide-loaded DCs at a responder to stimulator ratio of 5 : 1. After three rounds of weekly restimulation, the cultures were tested for the presence of SOX2-specific CTLs.

### Chromium release assay

Cytotoxic activity of the *in vitro*-stimulated CTLs was tested against T2 cells loaded with the individual SOX2-derived peptides or an irrelevant HLA-A^*^0201-binding peptide from HIV-1 reverse transcriptase at a concentration of 50 *μ*g ml^−1^, the glioma cell lines U343, U373, the primary GBM cell preparations DD-HT4 and DD-HT6559, the melanoma cell line 93.04A12. 1, and K562 cells in a 4 h standard ^51^Cr-release assay as described previously (Kiessling *et al*, 2002). The HLA-A2 restriction of CD8+T-cell-mediated lysis was evaluated at an effector cell to target cell ratio of 30 : 1 in the presence of the monoclonal anti-HLA-A2 antibody BB7.2 or an isotype-matched control antibody (BD Biosciences Pharmingen, Heidelberg, Germany) at a final concentration of 10 *μ*g ml^−1^.

## RESULTS

### Identification of *SOX2* as glioma-associated by analysis of DNA chip data and expression quantification by real-time PCR

**Figure 1:**
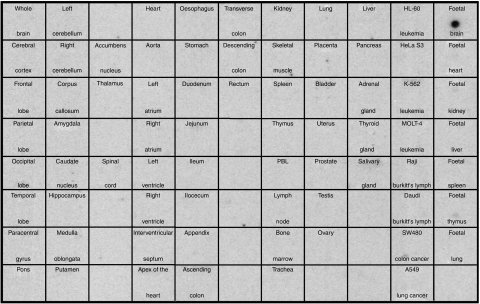
Dot blot analysis of *SOX2* mRNA expression pattern in normal tissues. A 112 bp cDNA fragment of the *SOX2* transcript was radioactively labelled and hybridised to the human MTE Array 2 that provides poly(A)^+^ RNA from 58 human adult tissues, eight tumour cell lines and seven human fetal tissues normalised to eight housekeeping genes. Strong hybridisation signals were revealed only in fetal brain. A1, whole brain; B1, cerebral cortex; C1, frontal lobe; D1, parietal lobe; E1, occipital lobe; F1, temporal lobe; F1, paracentral gyrus of the cerebral cortex; H1, pons; A2, cerebellum left; B2, cerebellum right; C2, corpus callosum; D2, amygdala; E2, nucleus caudatus; F2, hippocampus; G2, medulla oblongata, H2, putamen; B3, nucleus accumbens, C3, thalamus; E3, spinal cord; A4, heart; B4, aorta; C4, atrium left; D4, atrium right; E4, ventricle left; F4, ventricle, right; G4, interventricular septum; H4, apex of the heart; A5, oesophagus; B5, stomach; C5, duodenum; D5, jejunum; E5, ileum; F5, ilocaecum; G5, appendix; H5, colon ascendens; A6, colon transversum; B6, colon descendens; C6, rectum; A7, kidney; B7, skeletal muscle; C7, spleen; D7, thymus; E7, peripheral blood leucocyte; F7, lymph node; G7, bone marrow; H7, trachea; A8, lung; B8, placenta; C8; bladder; D8, uterus; E8, prostate; F8, testis; G8, ovary; A9, liver; B9, pancreas; C9, adrenal gland; D9, thyroid gland; E9, salivary gland; A10, leukaemia, HL-60; B10, HeLa S3; C10, leukaemia, K-562; D10, leukaemia, MOLT-4; E10, Burkitt's lymphoma, Raji; F10, Burkitt's lymphoma, Daudi; G10, colorectal adenocarcinoma, SW480; H10, lung carcinoma, A549; A11, fetal brain; B11; fetal heart; C11, fetal kidney; D11; fetal liver; E11, fetal spleen; F11, fetal thymus; G11, fetal lung.

By screening the Affymetrix DNA chip-based GeneExpress® database for genes overexpressed in GBM (Weigle *et al*, 2005), we identified the chip element 33109_f_at representing *SOX2*. The transcription factor *SOX2* was found to be overexpressed in the majority of GBM samples and other brain tumours, whereas expression in normal brain and other non-malignant tissues was almost negligible (data not shown). A 112 bp fragment of *SOX2* cDNA was radioactively labelled and subsequently hybridised to an MTE array representing pooled mRNA samples from 58 adult human tissues, eight human cell lines and seven fetal human tissues. Expression was detected exclusively in fetal brain (Figure 1), whereas all other tissues, especially normal adult brain, were *SOX2* negative.

**Figure 2:**
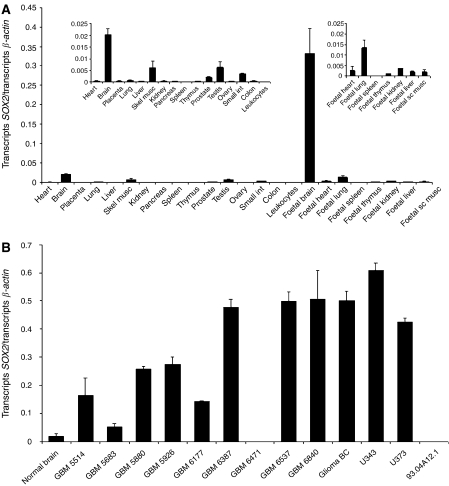
Real-time PCR analysis of *SOX2* expression in normal adult and fetal tissues and in brain tumours. (**A**) The tissue specificity of *SOX2* mRNA was determined by an SYBR Green I-based quantitative PCR assay in cDNA samples derived from 16 different pooled human adult tissues and eight different pooled human fetal tissues. The amount of *SOX2* transcripts was normalised to the quantity of transcripts of the housekeeping gene *β-actin*. High transcript levels of *SOX2* were found only in pooled fetal brain, whereas transcripts levels detected in adult brain, testis and skeletal muscle, the adult tissues with highest *SOX2* expression, were 16.5-, 52.9- and 54.1-fold lower, respectively (see insets). (**B**) The same assay was applied for the quantification of *SOX2* transcripts in brain tumours. *SOX2* was upregulated in almost all (nine out of 10) GBM samples, with eight of the samples displaying more than five-fold overexpression compared to pooled normal adult brain cDNA. The pooled adult brain sample from (**A**) is inserted for comparison. The sample marked ‘BC’ is commercially available from Biocat (see Materials and Methods). The results represent the means of two independent LC runs, bars indicate s.e. GBM, glioblastoma multiforme.

The expression of *SOX2* in different normal tissues and tumour specimens was thoroughly quantified with a sensitive real-time PCR method. Transcript quantities were determined in a panel of 16 human adult tissues representing major organs as well as in a panel of eight different fetal tissues (Figure 2A). To allow direct comparison of expression levels, the results were normalised to the transcript number of the housekeeping gene *β-actin*. Quantitative PCR verified high expression of *SOX2* in fetal brain (0.33 transcripts per *β-actin* transcript). In addition, minor expression in adult brain, skeletal muscle, testis and small intestine was detectable. The expression levels in these adult organs were 16.5-, 54.1-, 52.9- and 95.8-fold lower, respectively, when compared to fetal brain (Figure 2A, left inset). Regarding additional fetal organs, expression was highest in fetal lung and fetal kidney with 0.013 and 0.004 transcripts *SOX2* per *β-actin* transcript, respectively (Figure 2A, right inset).

To quantify *SOX2* expression in GBMs and in brain tumour cell lines, the amount of transcripts was determined in nine tumour specimens from GBM patients from the Department of Neurosurgery and in one commercially available sample from a malignant glioma patient by real-time PCR. In addition, the cell lines U343, U373 and 93.04A12.1 were tested for *SOX2* expression (Figure 2B). Expression of *SOX2* was upregulated in almost all (9 out of 10) samples as compared to cDNA from pooled normal brain. In 80% of the tumour specimens (8 out of 10), *SOX2* was overexpressed by more than five-fold, and in 40% of the samples (4 out of 10), *SOX2* was upregulated 20-fold or higher (Figure 2B). U343, U373 and the melanoma line 93.04A12.1 expressed 0.6102, 0.4258 and 0.0001 transcripts *SOX2* per transcript *β-actin*, respectively.

### Overexpression of SOX2 protein in tumour cells and GBM tissue

**Figure 3:**
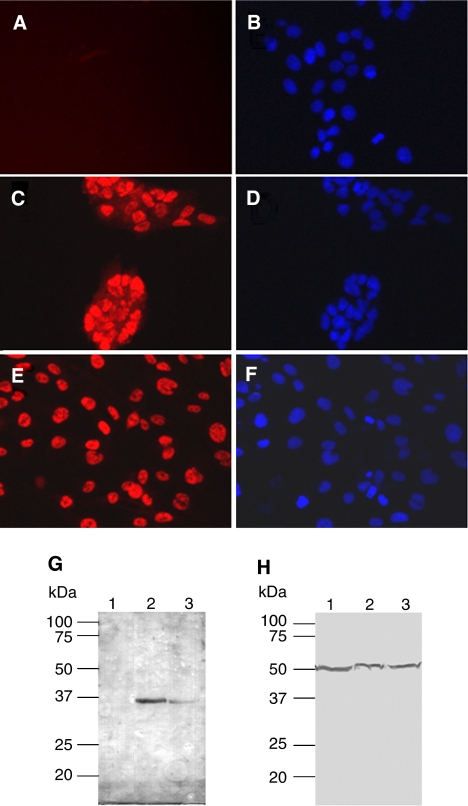
SOX2 protein expression in the glioma cell lines U343 and U373. Indirect immunofluorescence analyses of SOX2 expression. (**A**) 93.04A12.1 melanoma cells; (**C**) U343 and (**D**) U373 glioma cells. Predominant nuclear SOX2 expression was detected only in the glioma cells. (**B**, **D** and **F**) Appropriate counterstaining of cell nuclei with Hoechst33342. (**G** and **H**) Western blot analysis of total protein lysates from 93.04A12.1-melanoma cells (lane 1); U343 (lane 2) and U373 glioma cells (lane 3). (**G**) SOX2 was detected only in lysates of glioma cell lines. (**H**), equal loading of the gels was confirmed by using a monoclonal antibody against *α*-tubulin.

To confirm that the relative amount of *SOX2* mRNA is translated into appropriate protein levels, we performed indirect immunofluorescence analyses of U343 and U373 glioma cells as well as of 93.04A12.1-melanoma cells using a monoclonal anti-SOX2-antibody. As expected from the results of the quantitative mRNA analyses, SOX2 protein was not detected in 93.04A12.1-melanoma cells (Figure 3A and B) but was found predominantly in the nuclei of U343 and U373 glioma cells (Figure 3C–F). Additional Western blot analyses confirmed the expression of SOX2 in lysates of U343 and U373 cells (Figure 3G and H).

**Figure 4:**
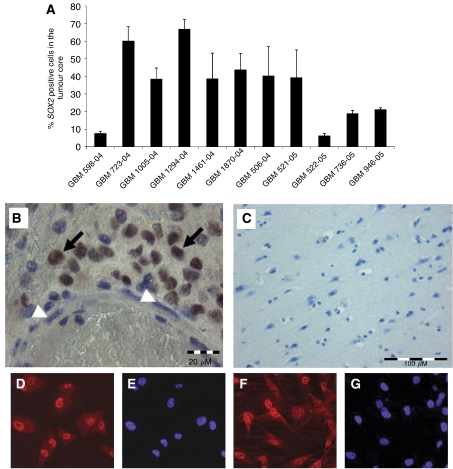
Immunohistochemistry of GBM tissue and primary GBM cells. Tumour cell nuclei positively stained for SOX2 protein by immunohistochemical analysis of glioblastoma tissue were quantified. Percentage of positive cells refers to the area of the compact tumour core and is the mean value (±s.e.m.) from three randomly selected high-power fields. (**A**) Overexpression of SOX2 protein in GBM can be detected by immunohistochemical analysis of tumour specimens. (**B**) In the neoplastic tissue, nuclei of tumour cells are intensely stained for SOX2 (arrows), whereas vascular structures of pathologic blood vessels remained unstained (arrowheads). (**C**) SOX2 staining is absent in normal cortex tissue from control cases without pathological findings. Bars=20 *μ*m in (**B**) and 100 *μ*m in (**C**). (**D**–**G**) The primary GBM cell lines DD-HT4 and DD-HT6559 express SOX2 protein. (**D** and **F**) SOX2 staining. (**E** and **G**) Counterstaining of nuclei using Hoechst33342.

Furthermore, we investigated GBM tissue from 11 patients for expression of SOX2 protein in tumour cells *in situ*, using immunohistochemistry. SOX2 was detected in all glioblastoma specimens. The number of positive tumour cells differed between individual cases, ranging from 6 to 66% in the central tumour core (Figure 4A). Independent of the percentage of positive cells, SOX2 appeared as strong staining, restricted to the nuclei of glioblastoma cells (Figure 4B). Other structures of the tumour, like blood vessels, were negative for SOX2. SOX2 could not be detected in cortex areas, which were not affected by tumour cell invasion, or in cortex tissue obtained from four control cases without brain tumours or other pathological findings in the brain (Figure 4C). The primary GBM cell preparations DD-HT4 and DD-HT6559 were positive for SOX2 protein expression (Figure 4D–G).

### Identification of a naturally processed T-cell epitope derived from SOX2

To investigate the potential of SOX2 as a target antigen for CTLs, the amino-acid sequence of SOX2 was screened for peptides predicted to bind to HLA-A^*^0201 representing the most frequent HLA-A allele in Caucasians by using the SYFPEITHI software (Table 1). The three highest scoring peptides fulfilling these criteria were synthesised and analysed for their binding affinity to HLA-A^*^0201 by a competition assay using peptide YLLPAIVHI from RNA helicase p72 as positive control and peptide ILK(FITC)EPVHGV from HIV-1 reverse transcriptase as reporter peptide. Binding affinities were classified as strong when the binding of a reporter peptide was inhibited by 75–100% with respect to inhibition of the reporter peptide binding by a positive control peptide, as intermediate when the inhibition was 50–74% and as weak when the inhibition was 25–49%. The peptides 60029 and 60030 bound with high affinity, whereas the peptide 60031 displayed a weak affinity (Table 1). All three peptides were used for the *in vitro* stimulation of CD8+ T lymphocytes.

**Figure 5:**
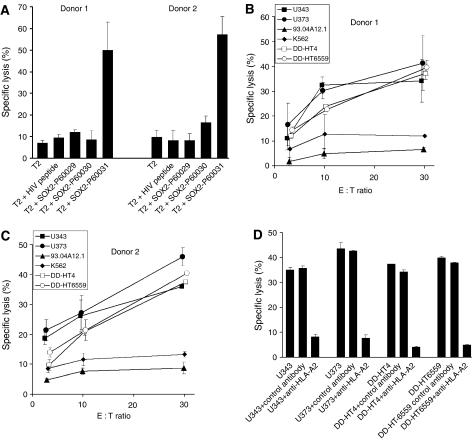
(**A**) *In vitro* generation of cytotoxic effector T cells specifically recognising the SOX2-derived peptide 60031. Purified CD8+ T lymphocytes of healthy donors were weekly stimulated by SOX2 peptide-pulsed autologous DCs. After four stimulations, T-cell cultures were tested for the activation of peptide-specific CTLs. The stimulated T cells were added to 3 × 10^3^ peptide-pulsed, ^51^Cr-labelled T2 target cells per well at an effector cell to target cell ratio of 20 : 1. Unloaded T2 cells and T2 cells pulsed with an irrelevant peptide from HIV reverse transcriptase served as controls. The results represent the mean values of triplicate determinations, bars indicate s.e.m. (**B** and **C**) SOX2-specific lysis of glioma cells by *in vitro*-generated cytotoxic effector cells. After four rounds of stimulation activated CD8+ T cells from the two donors were co-cultured with ^51^Cr-labelled U343, U373, DD-HT4, DD-HT6559, 93.04A12.1 or K562 tumour cells per well at various effector cell (E) to target cell (T) ratios (3 : 1, 10 : 1, 30 : 1). After 4 h of incubation, chromium release was determined. (**D**) HLA-A2-restricted recognition of glioma cells by SOX2 peptide-stimulated cytotoxic effector cells. Inhibition of T-cell-mediated cytotoxicity against U343, U373, DD-HT4 and DD-HT6559 cells was tested in the presence of a monoclonal anti-HLA-A2 antibody or an isotype-matched control antibody at an E : T ratio of 30 : 1. Columns represent mean±s.e.m. of results obtained from two different donors.

CD8+ T lymphocytes isolated from the blood of two healthy donors were weekly stimulated with autologous DCs pulsed with a cocktail of the peptides 60029, 60030 and 60031. After four stimulation cycles, T cells were tested for the presence of peptide-specific CTLs by chromium-release assays. Only peptide 60031 induced specific CTLs in both donors as shown by the specific lysis of T2 cells loaded with this peptide (Figure 5A). Unloaded T2 cells and T2 cells pulsed with a control peptide from HIV reverse transcriptase were only marginally lysed (Figure 5A). To determine whether peptide 60031 originates from intracellular processing of the SOX2 protein and is presented on the surface of tumour cells, the peptide-specific T cells were tested against the HLA-A2-positive glioma cell lines U343 and U373 expressing SOX2. The generated CTLs efficiently lysed cells of all glioma cell lines, whereas only marginal lysis was observed when the SOX2-negative melanoma cell line 93.04A12.1 expressing HLA-A2 was used as a control (Figure 5B and C). In addition, we analysed the cytotoxic activity of the CTLs against the SOX2-positive primary GBM cell preparations DD-HT4 and DD-HT6559 expressing HLA-A2 as determined by PCR and FACS analysis (data not shown). As demonstrated (Figure 5B and C), CTLs of both donors markedly lysed the primary GBM cell preparations. These results demonstrate the autochtonous generation and presentation of the peptide 60031 by glioma cells. Natural killer cell-like activity was excluded by the failure of the peptide 60031-activated T-cell populations to lyse K562 cells (Figure 5B and C). As illustrated in Figure 5D, the recognition of U343, U373, DD-HT4 and DD-HT6559 glioma cells was restricted to HLA-A2 as shown by a significant reduction of lytic activity in the presence of a monoclonal anti-HLA-A2 antibody.

## DISCUSSION

Immunotherapy represents a promising treatment option to improve the clinical outcome of patients suffering from malignant glioma. In this context, it has been documented that four out of 12 patients with newly diagnosed glioma who were treated with adoptive transfer of *ex vivo*-activated T lymphocytes showed partial regression of the tumour (Plautz *et al*, 2000). In patients with recurrent malignant gliomas, the local administration of *in vitro*-expanded tumour-reactive T cells resulted in complete or partial tumour regressions (Tsuboi *et al*, 2003). In addition, clinical trials revealed that DCs loaded with tumour surface-eluted peptides or tumour lysate efficiently augment tumour-reactive CTLs and intratumoral T-cell infiltration (Yu *et al*, 2001, 2004; Liau *et al*, 2005). Further data demonstrated that DC administration results in stable disease, minor or partial clinical responses as well as prolonged survival time (Yamanaka *et al*, 2003, 2005; Yu *et al*, 2004). Because activation of T cells against undefined mixtures of antigens generally bears the potential risk to induce destructive autoimmune reactions, the identification of well-characterised HLA class I binding peptides derived from glioma-associated antigens that serve as target structures for T cells is warranted.

However, the number of glioma-associated proteins known to elicit T-cell responses is rather limited. So far, T-cell epitopes derived from SART-1 and -3 (Imaizumi *et al*, 1999; Murayama *et al*, 2000), IL-13 receptor *α*2 chain (Okano *et al*, 2002), ADP-ribosylation factor 4-like (ARF4L) (Nonaka *et al*, 2002), UDP-Gal: betaGlcNAc beta1, 3-galactosyltransferase, polypeptide 3 (GALT3) (Tsuda *et al*, 2002), AIM-2 (Liu *et al*, 2004a), EphA2 (Hatano *et al*, 2005) and the type III variant of the epidermal growth factor receptor (EGFRvIII) (Wu *et al*, 2006) have been described. In addition, it has been shown that HER-2, gp100, MAGE-1 and TRP-2 were expressed in glioma and were recognised by CTLs (Liu *et al*, 2003, 2004b). However, the suitability of some candidates for specific immunotherapy of glioma is limited. Thus, ARF4L and GALT3 were markedly expressed in various normal tissues (Nonaka *et al*, 2002; Tsuda *et al*, 2002) bearing the potential risk of autoimmune reactions. In addition, gp100, MAGE-1, TRP-2 and EGFRvIII were detectable only in a part of gliomas restricting the number of potentially treatable patients (Chi *et al*, 1997; Liu *et al*, 2003, 2004b; Biernat *et al*, 2004). Owing to these limitations of several glioma-associated antigens and the heterogeneity of different types of malignant glioma (Chi *et al*, 1997; Nagane *et al*, 1997; Liang *et al*, 2005), the identification of additional target structures for CTLs is required.

In the present study, we demonstrated that the transcription factor *SOX2* is overexpressed in the vast majority of malignant gliomas, whereas expression in normal brain and other non-malignant tissues is almost negligible. The expression of SOX2 in glioma tissues, glioma cell lines as well as in the primary GBM cell preparations DD-HT4, and DD-HT6559 was also verified at the protein level. When investigating the suitability of SOX2 to serve as a target antigen for T cells, we identified an immunogenic HLA-A^*^0201-restricted peptide derived from SOX2, which proved to be effective in activating tumour-directed CTL responses. These results are in agreement with previous studies indicating the immunogenicity of several SOX family members. Thus, SOX1, SOX2, SOX3 and SOX21 as well as SOX4 were demonstrated to elicit humoral immune responses in small cell lung cancer patients (Gure *et al*, 2000; Friedman *et al*, 2004). In about one-third of glioma patients, Ueda *et al* (2004) could detect anti-SOX6 antibodies but so far, no T-cell epitope was described. The attractivity of SOX family members for T-cell-based immunotherapy of tumours was documented by a recent report indicating that SOX4 is overexpressed in lung carcinoma and can serve as a target structure of CTLs (Friedman *et al*, 2004). More recently, we identified SOX11 to be specifically overexpressed in the vast majority of malignant gliomas (Weigle *et al*, 2005) and demonstrated the generation of SOX11 peptide-reactive CTLs that were capable of lysing HLA-matched glioma cell lines (Schmitz *et al*, 2007).

In summary, we characterised the transcription factor SOX2 as a glioma-associated antigen that is abundantly and specifically overexpressed in these brain tumours. In addition, we identified an immunogenic HLA-A^*^0201-restricted T-cell epitope derived from SOX2 that effectively activated tumour-directed CTLs. Our results emphasise the suitability of this protein for a T-cell-based immunotherapy of glioma patients.

## Figures and Tables

**Table 1:** Prediction of HLA-A^*^0201-restricted SOX2-derived peptides and determination of binding affinities by a competition assay

**Peptide**	**Position^a^**	**Sequence**	**MW**	**Length^b^**	**Score**	**rBA (%)^c^**
60029	275–283	SMYLPGAEV	965.5	9	26	97.2
60030	131–139	LLAPGGNSM	858.4	9	25	80.0
60031	118–127	ALSPASSRSV	1239.7	10	24	43.3

Abbreviations: MW=molecular weight; rBA=relative binding affinity.

a The given numbers indicate the position of the peptide in the amino acid sequence of SOX2.

b Number of amino acids.

c The relative binding affinities were determined by comparing the inhibition of the reporter peptide binding by the analysed peptides in relation to the inhibition obtained with a positive control peptide, which was set as 100%. The positive control peptide was YLLPAIVHI from RNA helicase p72 and the reporter peptide was ILK(FITC)EPVHGV from HIV-1 reverse transcriptase. All peptides were used at a concentration of 10 *μ*M.
